# Comparison of Hourly PM_2.5_ Observations Between Urban and Suburban Areas in Beijing, China

**DOI:** 10.3390/ijerph121012264

**Published:** 2015-09-29

**Authors:** Ling Yao, Ning Lu, Xiafang Yue, Jia Du, Cundong Yang

**Affiliations:** 1 State Key Laboratory of Resources and Environmental Information System, Institute of Geographic Sciences and Natural Resources Research, Chinese Academy of Sciences, No.11A, Datun Road, Chaoyang, Beijing 100101, China; E-Mails: lexf@lreis.ac.cn (X.Y.); dujia@igsnrr.ac.cn (J.D.); 2 College of History and Tourism Culture, Inner Mongolia University, Hohhot 010021, China; E-Mail: nmgtour@126.com; 3 Jiangsu Center for Collaborative Innovation in Geographical Information Resource Development and Application, Nanjing 210046, China

**Keywords:** PM_2.5_ concentration, diurnal variations, spatial variations, day-of-week pattern, air quality

## Abstract

Hourly PM_2.5_ observations collected at 12 stations over a 1-year period are used to identify variations between urban and suburban areas in Beijing. The data demonstrates a unique monthly variation form, as compared with other major cities. Urban areas suffer higher PM_2.5_ concentration (about 92 μg/m^3^) than suburban areas (about 77 μg/m^3^), and the average PM_2.5_ concentration in cold season (about 105 μg/m^3^) is higher than warm season (about 78 μg/m^3^). Hourly PM_2.5_ observations exhibit distinct seasonal, diurnal and day-of-week variations. The diurnal variation of PM_2.5_ is observed with higher concentration at night and lower value at daytime, and the cumulative growth of nighttime (22:00 p.m. in winter) PM_2.5_ concentration maybe due to the atmospheric stability. Moreover, annual average PM_2.5_ concentrations are about 18 μg/m^3^ higher on weekends than weekdays, consistent with driving restrictions on weekdays. Additionally, the nighttime peak in weekdays (21:00 p.m.) is one hour later than weekends (20:00 p.m.) which also shows the evidence of human activity. These observed facts indicate that the variations of PM_2.5_ concentration between urban and suburban areas in Beijing are influenced by complex meteorological factors and human activities.

## 1. Introduction

Atmospheric particulate matter (PM), especially PM less than 2.5 μm in aerodynamic diameter (PM_2.5_), has recently been a concern of scientists and the public due to its quantified effects on human health, such as increased morbidity and mortality from cardiovascular and respiratory diseases, and even death rates [1,2,3,4]. Several studies carried out in Beijing have also demonstrated the relationships between high PM_2.5_ concentration and mortality and morbidity [5,6,7,8].

Beijing, the capital of China, with a population of over 20 million, is now facing a serious PM_2.5_ pollution problem. Along with the rapid economic development, the average concentration of PM_2.5_ in Beijing increased from 70–90 μg/m^3^ between 1989 and 1990 to 70–100 μg/m^3^ in 2000–2010 [9,10,11], about 2–3 times higher than the National Air Quality Standard (NAQS) and 5–6 times higher than the air quality guideline (AQG) recommended by the World Health Organization [12].

Moreover, it has been proved that high PM_2.5_ concentration leads to the occurrence of haze [13,14]. Sensitivity test shows that the mass concentration threshold of PM_2.5_ to cause haze occurrence was about 80 μg/m^3^ when the relative humidity was 70% in Beijing [15]. Lower visibility occurred mainly in the urban areas of Beijing, where the number of haze days showed an increasing trend [16]. Unfortunately, Beijing experienced more than 160 haze days in 2013 according to the government report. Consequently, identifying the spatial and temporal variations in PM_2.5_ concentration would have immense significance to make effective pollution control strategy [17].

Numerous studies about spatial and temporal variations of PM_2.5_ have been carried out in the United States and Europe [18,19,20,21,22,23]. These studies demonstrated the PM_2.5_ pollutions in urban areas were more severe than suburban and the rural areas. In Beijing, most of the related studies about PM_2.5_ focused on PM_2.5_ chemical compositions and their correlations or sources [24,25,26,27] and paid little attention to the differences between urban and suburban areas. In additional, some studies put emphasis on seasonal and diurnal variations of PM_2.5_ in Beijing [11,28,29]. These studies pronounced that seasonal and diurnal variations in PM_2.5_ concentration are found at both urban and rural stations, while higher PM_2.5_ concentrations occurred in the urban areas almost in all seasons [30]. However, these works were based on only one or two stations. The objective of this study is to explore the spatial and temporal variations of PM_2.5_ mass concentration in Beijing. Hourly PM_2.5_ concentration observations were collected at 12 stations over a 1-year period to identify the spatial and temporal variations between urban and suburban areas. To explore the spatial clustering and general temporal characteristics of PM_2.5_ mass concentration at different locations, we clustered the observation stations into different categories with K-means method. The cluster centers are analyzed to compare their spatial and temporal variations.

The rest of the paper is organized as follows: Materials and methods used are introduced in Section 2. Results and discussion of interesting spatial and temporal variations of PM_2.5_ concentration between urban and suburban areas are presented in Section 3. Finally, we present our conclusions in Section 4.

## 2. Material and Methods

### 2.1. Data Description

Hourly PM_2.5_ observations of 12 air pollution monitoring stations within Beijing City obtained from the China Environmental Monitoring Center (CEMC, http://113.108.142.147:20035/emcpublish/) during the period 1 March 2013 to 28 February 2014 were collected. These observations are measured by continuous particulate monitor with Tapered Element Oscillating Microbalances (TEOM) or beta-attenuation method [31,32]. Additionally, PM_2.5_ concentrations during the same period at the U.S. embassy station are also prepared to verify the monthly variation. Since the U.S. embassy station uses a different sampling instrument and method from the CEMC stations, PM_2.5_ concentration data collected at the U.S. embassy station is used as a reference value. 

CEMC defines the 12 stations as eight urban assessment stations and four suburban assessment stations (Table 1). Because the spatial distributions and the surrounding environments of these stations are different, the observations reflect different elevations and a mix of station surroundings [20]. Since the monitoring network has been deployed incrementally over the last three years, the period of record varies from monitoring station to monitoring station. Station 1012 was established approximately one month later than other stations and lacked the data for March, so in subsequent analyses, station 1012 was excluded in the station clustering step, while analyses on temporal variation utilized all available data.

**Table 1:** Codes, names and categories of the 12 stations.

Code	Name	Category
1001	Wanshouxigong	Urban
1002	Dingling	Suburban
1003	Dongsi	Urban
1004	Tiantan	Urban
1005	Nongzhanguan	Urban
1006	Guanyuan	Urban
1007	Wanliu	Urban
1008	Shunyi	Suburban
1009	Huairou	Suburban
1010	Changping	Suburban
1011	Aoti	Urban
1012	Gucheng	Urban

### 2.2. K-Means Clustering Method

K-means method is used because it can map the original data to a higher dimensional feature space so that the data can be easily separated linearly [17]. K-means is a clustering method aims to partition nonlinearly and high-dimensional separated observations into appropriate clusters. For clustering a given set of PM_2.5_ observations (*x*_1_,*x*_2,_…,*x_n_*) into *k* sets (*k* ≤ *n*), that is, *S* = {*s*_1_,*s*_2_, …,*s_k_* }, the best appropriate partition is determined with the minimum within-cluster sum of squares (WCSS) which means the sum of the squared Euclidean distances between each observation and the corresponding cluster center [17]. WCSS can be described as Equation (1):

(1) $$WCSS=\sum_{i=1}^{k}\sum_{x_{j}\in s_{i}}||X_{j}-\mu_{i}|{|}^{2}$$

where μ*_i_* is the average dissimilarity of sample *i* to all other stations in cluster *k*; and *X_j_* stands for PM_2.5_ observations at each station. For a given cluster number *k*, the algorithm proceeds by alternating between following two steps [33]:

- assign each PM_2.5_ observation to the cluster whose mean yields:
  (2) $$S_{i}^{\left(t\right)}=\{x_{p}:||x_{p}-m_{i}^{\left(t\right)}|{|}^{2}\leq ||x_{p}-m_{j}^{\left(t\right)}|{|}^{2}\forall j,1\leq j\leq k\}$$
- calculate the new means to be the centroids of the PM_2.5_ observations in the new clusters:
  (3) $$m_{i}^{\left(t+1\right)}=\frac{1}{\left|S_{i}^{\left(t\right)}\right|}\sum_{x_{j}\in S_{i}^{\left(t\right)}}X_{j}$$
- repeat steps a and b until WCSS is stable, which means the difference between adjacent iterations is less than a small threshold.

The correct cluster number *k* is determined with silhouette method which is first described by Rousseeuw [34]. Suppose the number of samples in cluster r is nr ($n_{r}=|C_{r}|$). The silhouette coefficient is defined as:

(4) $$S(i)=\frac{b(i)-a(i)}{\max\{a(i),b(i)\}}$$

(5) $$a(i)=\frac{1}{n_{r}}\sum_{x_{j}\in C_{r}}d(x_{i},x_{j})$$

(6) $$b(i)=\min(\frac{1}{n_{k}}\sum_{x_{j}\in C_{k}}d(x_{i},x_{j}))$$

where, S(*i*) is the silhouette of PM_2.5_ observation *i*; a(*i*) is the average dissimilarity of sample *i* to all other stations in cluster *r*; and b(*i*) is the least average dissimilarity of PM_2.5_ observation *i* to the stations within a cluster different from cluster *r*. Thus, a smaller S(*i*) value indicates a better similarity among stations within the same cluster. The overall quality of a clustering distribution can then be measured using the average silhouette width for the entire PM_2.5_ concentration data set, which is defined as:

(7) $$SC=\frac{1}{n}\sum_{i=1}^{n}S(i)$$

where *n* is the total number of PM_2.5_ observations. A higher value of SC indicates better discrimination among clusters of a mining result. The k value, as maximized by the SC, was selected as the final PM_2.5_ cluster number.

However, it is possible for k-means to reach a local minimum because of the starting points. We perform number of times to repeat the clustering, each with a new set of initial cluster centroid positions. K-means returns the solution with the lowest value for *SumD* (Equation (8)), and this solution is selected as the final PM_2.5_ cluster result:

(8) $$SumD=\sum_{k=1}^{K}\sum_{x_{i}\in C_{k}}d(x_{i}-{\bar{x}}_{k}),{\bar{x}}_{k}=\frac{1}{n_{k}}\sum_{x_{i}\in C_{k}}x_{i}$$

## 3. Results and Discussion

### 3.1. Annual PM_2.5_ Concentration

Taking all of the 12 stations into account, the annual arithmetic mean PM_2.5_ concentration during the research period is about 87 μg/m^3^ (Table 2). A large difference is found between the average concentrations for the cold and warm seasons, 105 and 77 μg/m^3^, respectively. In this study, it is estimated that approximately 174 days of the daily PM_2.5_ concentrations exceeded the daily PM_2.5_ standard of NAQS (75 μg/m^3^) during the 1-year research period.

**Table 2:** Summary statistics for PM_2.5_ concentrations.

Season	Mean	Standard Deviation	Max
Year	87	80	623
Spring	85	73	515
Summer	80	68	560
Autumn	77	73	376
Winter	105	100	623

### 3.2. Seasonal Patterns of PM_2.5_ Concentration

**Figure 1:**
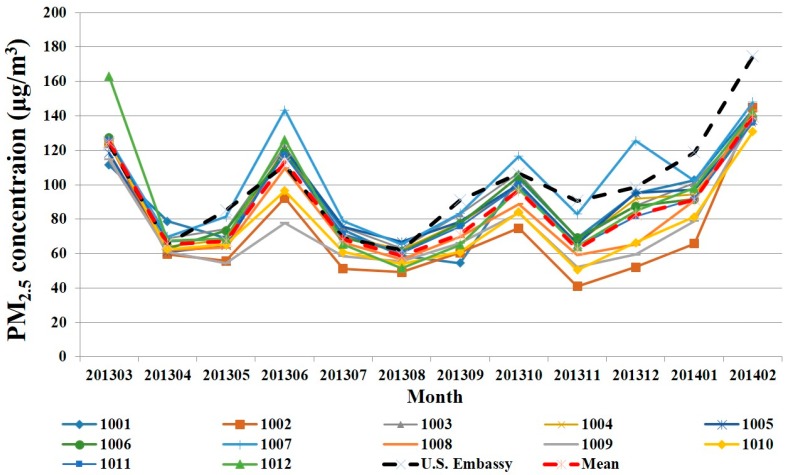
All stations demonstrate a similar seasonal pattern of PM_2.5_ concentration. Though the sampler and sampling precision are different, the U.S. embassy station is used as a reference value to verify the monthly variation.

A seasonal pattern is evident at each sampling station, which shows very little variability in the monthly average scale (Figure 1). Furthermore, sampling data at the U.S. embassy station of the same period also indicates a similar monthly variation. For individual months, it is found that Beijing has four significant peaks in March 2013, June 2013, October 2013, and February 2014. The highest level (around 140 μg/m^3^) appears in February 2014, and November 2013 is at the minimum. Station 1007 is a special case, as the traffic flow around this station is always larger than those of surrounding stations; it consequently has higher PM_2.5_ concentrations than other surrounding stations in each month, and its lowest value appears in August 2013 (about 65 μg/m^3^). Likewise, it is also evident that a seasonal cycle for individual days of the week appears the similar situation based on the PM_2.5_ daily concentration (not shown).

To our knowledge, such a seasonal pattern of PM_2.5_ concentration in Beijing is unique as compared with other major cities. For instance, New York shows a marked peak in July, with minimum PM_2.5_ concentrations occurring in February [20], while Athens (Greece) demonstrates a bimodal pattern (March and December) [19].

### 3.3. Stations Variations

The Pearson correlation coefficient (R) and distance between every two stations are investigated to verify the inter-correlations of all 12 stations (Table 3). Most of R values between every two stations are greater than 0.9, especially when the distance between the two stations are less than 5 kilometers (station 1003, 1004, 1005, and 1006), R exceeds 0.95. However, we find that correlations between urban and suburban stations are mostly less than 0.85. Consequently, it is necessary to cluster the stations before studying the spatial distribution pattern of PM_2.5_ concentration in Beijing.

**Table 3:** Inter-correlations of PM_2.5_ observations at all stations. R is the Pearson correlation coefficients, and D stands for the distance between every two stations, the unit is kilometers.

	R	1001	1002	1003	1004	1005	1006	1007	1008	1009	1010	1011	1012
D													
1001		1	**0.79**	0.93	0.94	0.93	0.93	0.90	0.88	0.82	0.84	0.92	0.92
1002		**47.38**	1	0.83	0.84	0.83	0.85	0.85	0.85	0.89	0.94	0.85	0.85
1003		7.93	43.7	1	0.97	0.97	0.97	0.93	0.90	0.84	0.87	0.96	0.91
1004		4.78	47.87	4.86	1	0.97	0.96	0.93	0.90	0.84	0.88	0.95	0.91
1005		11.38	44.48	3.86	7.31	1	0.95	0.92	0.91	0.84	0.87	0.96	0.91
1006		5.78	41.61	6.65	7.52	10.44	1	0.95	0.91	0.85	0.89	0.97	0.94
1007		13.33	34.39	12.82	15.19	15.84	7.83	1	0.90	0.85	0.89	0.95	0.94
1008		37.85	41.24	29.92	34.12	26.82	34.77	34.89	1	0.90	0.87	0.92	0.89
1009		55.27	34.83	47.86	52.62	45.74	50.72	47.72	22.47	1	0.90	0.87	**0.85**
1010		39.10	8.38	35.76	39.77	36.82	33.34	26.03	37.47	35.95	1	0.89	0.90
1011		12.18	37.61	6.13	10.71	7.4	7.69	9.39	27.24	43.19	29.74	1	0.93
1012		14.88	42.14	19.94	19.28	23.76	13.32	11.96	46.58	**59.54**	33.92	19.67	1

### 3.4. Spatial Distribution of Station Clustering

**Figure 2:**
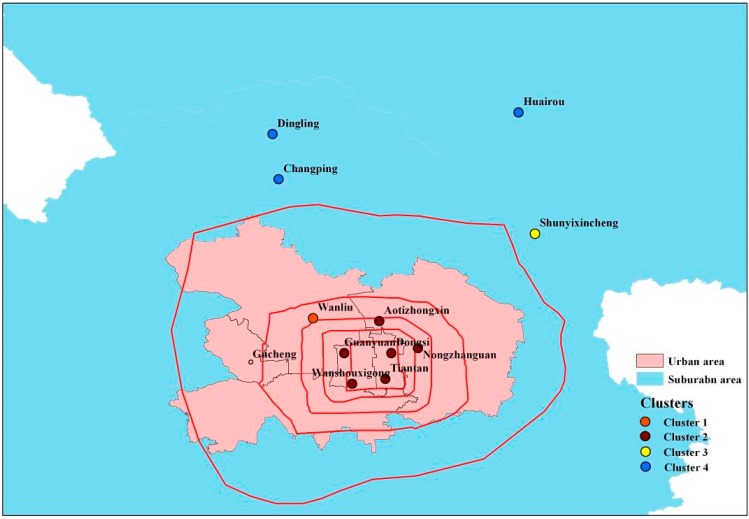
K-means clusters for urban and suburban stations. Stations are clustered into four categories. Cluster 1 and 2 locate in urban and the others locate in suburban. The average PM_2.5_ concentration decreases from cluster 1 to cluster 4. The red lines represent the six ring roads in Beijing.

K-means method is used to cluster the 11 stations (station 1012 being excluded) into suitable categories, intending to filter the strong seasonal, diurnal and, to a lesser degree, day-of-the-week effects from the subsequent spatial analysis. We calculate SC indexes for different cluster numbers from 1 to 7, and find that it reaches a maximum when the cluster number is 4, indicating that the between-cluster dissimilarity is large while the within-cluster dissimilarity is small. So the 11 stations are partitioned into four clusters (Figure 2).

The first cluster is the single station 1007. The second cluster contains six stations: stations 1001, 1003, 1004, 1005, 1006, and 1011. Station 1008 belongs to the third cluster. The fourth cluster contains three stations: stations 1002, 1009, and 1010. The first-cluster locates in the north-west of urban, between the fourth-ring road and the fifth-ring road; stations of the second cluster mainly locate inside the fourth-ring road of Beijing City, south-east of urban. The third-cluster and fourth-cluster stations are outside the sixth-ring road, in suburban area. The first cluster has the most severe pollution among the four clusters with the largest average PM_2.5_ concentration, while the degree of pollution in the fourth cluster is the least. Hu *et al.* clustered the stations into three categories with PM_10_ concentration [17], compared with PM_10_, PM_2.5_ indicates a different situation. The most distinguished difference lies in station 1007; it belongs to the second cluster in PM_10_ clustering but is separated as an individual cluster in PM_2.5_. This phenomenon may be interpreted by the different source of PM_10_ and PM_2.5_.

### 3.5. Diurnal Patterns of PM_2.5_ Concentration

**Figure 3:**
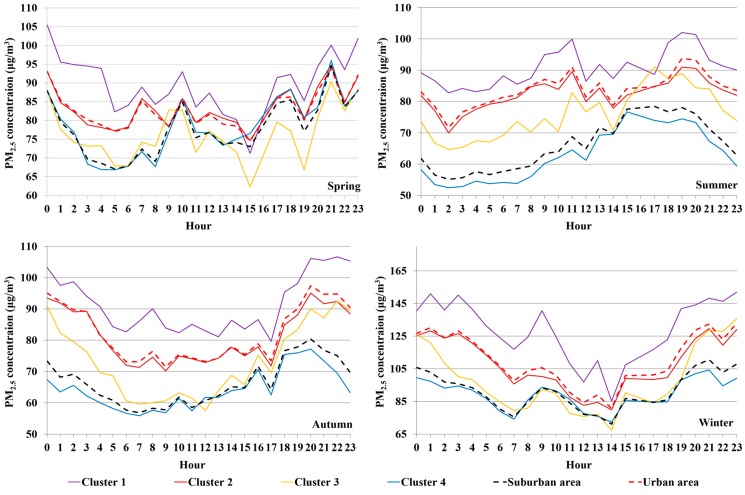
Similar diurnal pattern of PM_2.5_ concentration at all clusters. It is clear that urban areas have higher PM_2.5_ concentration than suburban. Cluster 1 always suffers the highest PM_2.5_ concentration and cluster 4 mostly enjoys the minimum value.

As shown in Figure 3, a pronounced diurnal cycle in PM_2.5_ concentrations is evident among all the clusters. On the whole, the diurnal variation of PM_2.5_ is observed with higher concentration at night than daytime, the minimum concentration generally appears in the early afternoon. This finding is consistent with prior research [28]. It is worth noting that there is a significant minimum around noon (14:00 p.m.), because the meteorological condition at this time is impeditive for the formation of the thermal inversion layer.

Urban areas suffer higher PM_2.5_ concentration (about 92 μg/m^3^) than suburban areas (about 77 μg/m^3^), and PM_2.5_ concentration of cluster 1 is always much higher than cluster 2 while cluster 3 gets higher PM_2.5_ concentration than cluster 4. That is, north-west area has higher PM_2.5_ concentration than east area in urban. For suburban areas, north-east area (in Shunyi Distinct) near urban suffers higher PM_2.5_ concentration than other suburban areas. These facts indicate the influence of complex human activities on PM_2.5_ concentration.

Nevertheless, the diurnal pattern of PM_2.5_ for all clusters presents systematic seasonal variations. In spring, the evening rush hour peak appears between 19:00 p.m. and 21:00 p.m., followed by a 22:00–24:00 p.m. peak, while it rises at 18:00 p.m. in winter. In summer, the evening rush hour peak is 17:00 p.m.–19:00 p.m. followed by a notable decline. These observations indicate the mobile-source influence on PM_2.5_. The major difference is that PM_2.5_ concentrations rise at 22:00 p.m. in winter, while the opposite situation appears in summer and autumn. This may be related with the subsidence inversion at night in winter, as a result the stability of the atmosphere increases and the pollutions cannot easily spread around. And this weather condition hardly ever appears in summer or autumn.

In both urban and suburban areas, the morning rush hour peak is observed in spring (8:00 a.m.–10:00 a.m.) and winter (7:00 a.m.–9:00 a.m.), but not apparent in summer and autumn. The evening rush hour peak is notable in spring (19:00 p.m.–21:00 p.m.), autumn (17:00 p.m.–20:00 p.m.), and winter (18:00 p.m.–21:00 p.m.). There is a special case in summer, urban areas show an evening rush hour peak (18:00 p.m.–20:00 p.m.) while suburban areas do not show it. This could suggest that enhanced anthropogenic activity is not solely responsible for the rush hour PM_2.5_ peak and the cumulative growth of nighttime PM_2.5_ concentration in summer maybe due to the atmospheric stability. Also, it indicates that the rush hour peak doesn’t occur near the time when atmospheric stability is normally at a maximum. Theoretically, if the rush hour peak always occurs near the time when atmospheric stability is normally at a maximum, the time of occurrence of the nighttime peak in winter should be earlier than that in summer, but in fact quite the opposite.

### 3.6. Day-of-Week Pattern

**Figure 4:**
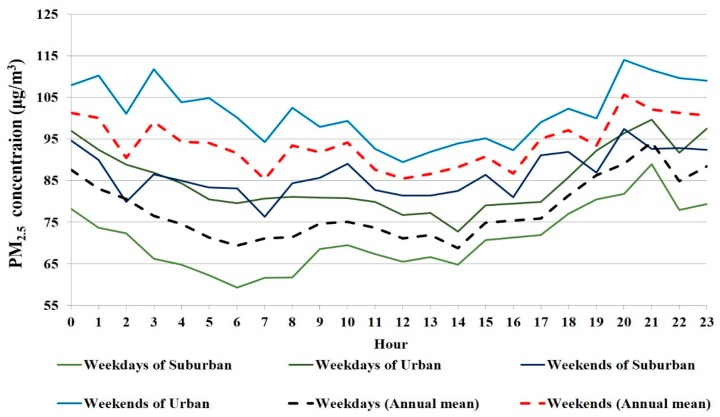
Day-of-week pattern in urban and suburban areas. Though urban concentration is higher than suburban in both weekdays and weekends, the day-of-week patterns are similar. Interestingly, the evening peak is an hour later in weekdays than weekends, reflecting human activities.

In general, yearly average PM_2.5_ concentration is about 18 μg/m^3^ (about 21% of the annual mean concentration) higher on weekends (Saturdays and Sundays) than on weekdays (Figure 4). This phenomenon is probably due to the driving restriction in Beijing, that is, about 20% of cars have to stay off the road on each weekday. Since the vehicle possession level amounts to close to 5.35 million units, this means there will be 1.07 million more cars on the road on weekends than weekdays. Supposing the other emissions are constant, the sudden increase of cars on the road might be the main cause of the higher PM_2.5_ concentration at weekends.

Furthermore, urban PM_2.5_ concentrations are higher than suburban PM_2.5_ concentrations on both weekdays and weekends. The nighttime peak of weekdays (21:00 p.m.) is one hour later than weekends (20:00 p.m.) in either urban or suburban areas; this also results from human activity. By contrast, there is a totally different situation in other big cities. Taking New York for example, PM_2.5_ concentrations across the city are significantly lower on weekends and uniformly high on weekdays [20].

Besides the interesting facts above, we also find some phenomena that cannot be easily explained by atmospheric condition or emission, mobile-source influence. For example, the unique seasonal pattern of PM_2.5_ concentration in Beijing, and PM_2.5_ concentrations start to rise from 2:00 a.m. until 11:00 a.m. in summer while it begins falling at 0:00 a.m. until 5:00 a.m. in other seasons. Therefore, further studies should be carried out with multi-resources data, such as pollution source information, real-time population grid data, meteorological data and traffic data, to provide reasonable interpretations for these unexplainable phenomena.

## 4. Conclusions

The spatial and temporal characteristics of PM_2.5_ concentration in Beijing City between March 1, 2013 and February 28, 2014 are analyzed based on the hourly observations at 12 stations. On the whole, the annual mean PM_2.5_ concentration indicates large differences between urban and suburban as well as cold and warm seasons in Beijing.

K-means method is involved to cluster the stations into four categories for diurnal and day-of-week patterns analysis. The diurnal variation of PM_2.5_ is observed with higher concentration at night and lower value at daytime, and the cumulative growth of nighttime PM_2.5_ concentration maybe due to the atmospheric stability. We also find that PM_2.5_ concentrations are about 18 μg/m^3^ (about 21% of the annual mean concentration) higher on weekends than the other days of the week, which might be related with the driving restrictions implemented in Beijing. The nighttime peak in weekdays (21:00 p.m.) is one hour later than weekends (20:00 p.m.) which also shows the evidence of human activity.

These are still some phenomena cannot be easily explained by atmospheric condition or emission, mobile-source influence, but may be helpful for the air quality model to exploit a deep understanding of pollution mechanism and improve its simulation accuracy.
